# Reducing Water Absorption and Improving Flexural Strength of Aluminosilicate Ceramics by MnO_2_ Doping

**DOI:** 10.3390/ma17112557

**Published:** 2024-05-25

**Authors:** Bingxin Yang, Shaojun Lu, Caihong Li, Chen Fang, Yan Wan, Yangming Lin

**Affiliations:** 1 College of Chemistry and Materials Science, Fujian Normal University, Fuzhou 350117, China; xmyangbingxin@fjirsm.ac.cn; 2 Xiamen Key Laboratory of Rare Earth Photoelectric Functional Materials, Xiamen Institute of Rare Earth Materials, Haixi Institute, Chinese Academy of Sciences, Xiamen 361021, China; xmlushaojun@fjirsm.ac.cn (S.L.); xmlicaihong@fjirsm.ac.cn (C.L.); xmfangchen@fjirsm.ac.cn (C.F.); 3 Fujian Institute of Research on the Structure of Matter, Chinese Academy of Sciences, Fuzhou 350002, China

**Keywords:** MnO_2_, aluminosilicate ceramics, water absorption, porosity, flexural strength

## Abstract

As key performance indicators, the water absorption and mechanical strength of ceramics are highly associated with sintering temperature. Lower sintering temperatures, although favorable for energy saving in ceramics production, normally render the densification degree and water absorption of as-prepared ceramics to largely decline and increase, respectively. In the present work, 0.5 wt.% MnO_2_, serving as an additive, was mixed with aluminosilicate ceramics using mechanical stirring at room temperature, achieving a flexural strength of 58.36 MPa and water absorption of 0.05% and lowering the sintering temperature by 50 °C concurrently. On the basis of the results of TG-DSC, XRD, MIP, and XPS, etc., we speculate that the MnO_2_ additive promoted the elimination of water vapor in the ceramic bodies, effectively suppressing the generation of pores in the sintering process and facilitating the densification of ceramics at a lower temperature. This is probably because the MnO_2_ transformed into a liquid phase in the sintering process flows into the gap between grains, which removed the gas inside pores and filled the pores, suppressing the generation of pores and the abnormal growth of grains. This study demonstrated a facile and economical method to reduce the porosity and enhance the densification degree in the practical production of aluminosilicate ceramics.

## 1. Introduction

The porosity of ceramic materials highly depends on the sintering process and the sintering temperature as key parameters, and is closely related to the population, shape, and distribution of pores in ceramics, thereby affecting the performance of ceramics, e.g., the mechanical strength and water adsorption, etc. [1,2]. Higher sintering temperatures favor gas diffusion in ceramic materials to suppress the formation of pores [3]. Due to the complex crystallization properties of aluminosilicate ceramic oxide components, high temperatures (>1200 °C) are normally employed to ensure complete sintering [4,5,6], giving rise to huge energy consumption.

Recently, the implementation of energy-saving production policies have motivated many industries, especially high energy-consuming industries, to develop new technologies to reduce energy consumption [7,8,9]. For the ceramic industry, the key point is to lower the sintering temperature while ensuring sufficiently high mechanical strength [10,11] and low water absorption [12,13,14] to meet the production requirements.

In the past few years, some fundamental studies have discovered that the modification of a single component of common ceramic oxides, i.e., the introduction of additives, can theoretically suppress the growth of grains and induce grain refinement, thus enhancing the strength and toughness of ceramics to a certain extent and ultimately achieving a reduction in sintering temperature [15,16,17,18]. For instance, Wang et al. [19] investigated the microstructural evolution of Al_2_O_3_ with different Nd_2_O_3_ additions, showing that the addition of neodymium inhibits the densification of Al_2_O_3_ through grain boundary segregation. In a similar study, Hassan’s team [20] reported the effect of the Nb_2_O_5_ amount on the densification and mechanical properties of Al_2_O_3_, and revealed that even a small amount of Nb_2_O_5_ (0.6 vol.%) can improve the densification, bending strength, hardness and fracture toughness of the Al_2_O_3_ matrix, attributed to the decrease of porosity and the strengthening of the Nb_2_O_5_ crystal interface. These results demonstrated the feasibility that doping with additives is capable of densifying the ceramic materials and lowering the sintering temperature concurrently. Of note, the local brittleness caused by the change in stress orientation in the sintering process and the difficulty in reducing the porosity are still the biggest challenges confronted in practical ceramic production.

Adding sintering aids, such as MgO or ZrO_2_, into the alumina ceramic, makes it able to suppress the growth of grains, thereby largely preventing more pores from generating [21,22]. Furthermore, some liquid phase sintering aids which transform into a liquid phase at the stage of high-temperature sintering, e.g., SiO_2_ and CaO, can lower the sintering temperature of alumina ceramics, together with reducing the mechanical strength [6,23,24,25]. Moreover, aluminosilicate ceramics made from geologic minerals are less costly than SiO_2_-doped alumina ceramics [26,27,28,29,30] and are thus extensively used in various fields, particularly construction materials and daily ceramics, both of which desire to achieve a low-consumption production of ceramics with high flexural strength and low water absorption properties [31].

In the present work, to further reduce the production cost while ensuring the flexural strength and water absorption remain undegraded, MnO_2_ was employed as a sintering aid. Aluminosilicate ceramics with 0~0.5 wt.% MnO_2_ addition were sintered at 1150 °C, and the impact of Mn doping on the flexural strength, porosity, and water absorption of the ceramics was characterized and disclosed.

## 2. Materials and Methods

### 2.1. Materials

MnO_2_ was purchased from Innochem, Beijing, China (Mn 58% min.). The aluminosilicate ceramic slurry used in this experiment was provided by Liangci Technology Co., Ltd. (Quanzhou, China), with a content of 356 g/200 mL. The chemical composition of raw materials is shown in Table 1.

### 2.2. Preparation of MnO_2_-Doped Aluminosilicate Ceramic

The MnO_2_-doped aluminosilicate ceramics were prepared using mechanical agitation at room temperature and calcination. Typically, after the provided aluminosilicate ceramic slurry was mechanically stirred for 4 h at 400 rpm, a certain amount of MnO_2_ (0, 0.05, 0.1, 0.3, 0.5 wt.%) was added to the uniformly dispersed slurry, followed by stirring for another 12 h at 800 rpm to achieve thorough mixing of MnO_2_ and ceramic slurry. Then, the mixture was poured into a plaster mold and left for 2 h. After the plaster mold was removed, the sample was dried for 12 h at 55 °C and for another 4 h at 110 °C. The obtained sample (ceramic body) was cooled down in a desiccator and denoted as M-0, M-0.05, M-0.1, M-0.3, and M-0.5, respectively.

The obtained ceramic body was sintered at the desired temperature (1150 °C or 1200 °C) for 8 h, and the period of elevating temperature was set as 12 h. According to the MnO_2_ amount and sintering temperature, the finally obtained samples were denoted as M-0-1200 (as control sample), M-0-1150, M-0.05-1150, M-0.1-1150, M-0.3-1150, and M-0.5-1150, respectively.

### 2.3. Measurements and Characterization

#### 2.3.1. Water Absorption Measurement

To begin with, the sintered sample was rammed to a suitable size and weighed (the mass was denoted as m_1_). Subsequently, the sample was immersed in boiling water for 2 h and soaked in water at room temperature for 20 h, followed by removing the surface moisture with a wet towel and weighing (the mass was denoted as m_2_). The water absorption was calculated using the following equation:

(1) $$W=\frac{m_{2}-m_{1}}{m_{1}}\text{×}100\%$$

where W(%) is the water absorption, $m_{1}\;$(g) is the mass of dry sample, and $m_{2}\;$(g) is the mass of wet sample.

#### 2.3.2. Flexural Strength Measurement

The flexural strength of the ceramic was measured using the universal material testing machine (2365, INSTRON, Norwood, MA, USA). The indenter contacted the upper surface of the sample via manual adjustment, and the movement rate of the upper indenter was 0.5 mm min^−1^. The measurement for each sample was repeated three times, and the average value was provided. The flexural strength was calculated using the following equation:

(2) $$\sigma_{f}=\frac{8\mathrm{FL}}{\pi R^{3}}$$

where $\sigma_{f}$ (MPa) is the flexural strength, F (N) represents the load when the sample breaks, L (mm) is the span (100 mm), and R (mm) is the radius of the sample section.

#### 2.3.3. Characterization

Mercury intrusion porosimetry (MIP) was carried out using the mercury injection apparatus (AutoPore W9510, Micromeritics, Norcross, GA, USA) applicable for the aperture measurement range of 5 nm~800 μm. The unsintered ceramic sample was subjected to qualitative and quantitative analysis using the thermogravimetry-differential scanning calorimetry (TGA/DSC 1, Mettler Telodo, Zurich, Switzerland). The sample (20 mg) was heated from 30 to 1200 °C with a heating ramp of 10 °C min^−1^; the heating rate was 10 °C min^−1^. The component contents of the materials were qualitatively determined using the X-ray fluorescence (XRF) spectrometer (Axios, Panalytical, Almelo, The Netherlands). The composition of the sintered ceramics was analyzed using the X-ray diffractometer (XRD) (Miniflex 600, Rigaku, Akishima-shi, Japan). The sample was measured using the scanning speed of 10° min^−1^ with a step size of 0.02°. X-ray photoelectron spectroscopy (XPS) analysis was performed on X-ray photoelectron spectroscopy system (K-Alpha, Thermo Fisher Scientific, Waltham, MA, USA) equipped with Al Kα X-ray radiation as the X-ray source. Fourier transform infrared spectroscopy (FTIR) (Nicolet iS 50, Thermo Fisher Scientific, MA, USA) was performed in the range 4000–400 cm^−1^. The morphology and chemical composition of the ceramics were obtained using a field emission scanning electron microscope (SEM) (Apreo S LoVac, Thermo Fisher Scientific, MA, USA) equipped with Energy dispersive X-ray spectrometer (EDS).

## 3. Results and Discussion

### 3.1. Phase Analysis

**Figure 1:**
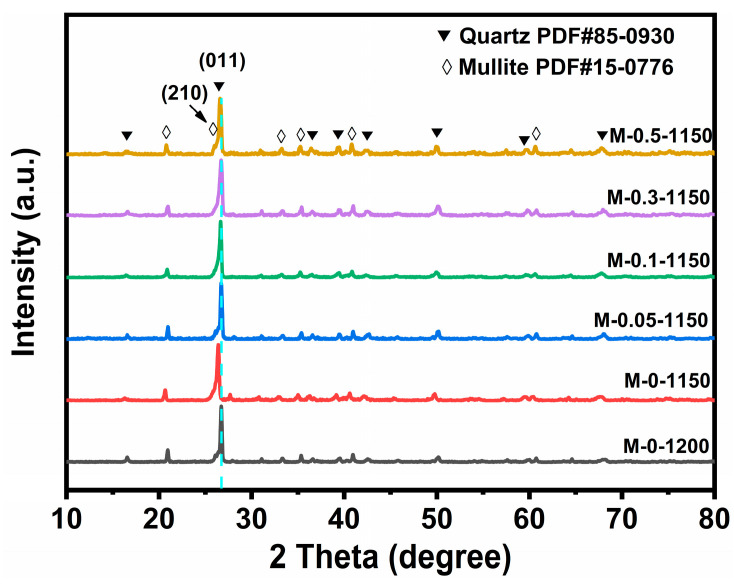
XRD patterns for M-0-1200, M-0-1150, M-0.05-1150, M-0.1-1150, M-0.3-1150 and M-0.5-1150.

Figure 1 shows the XRD patterns for the six ceramic samples prepared at different conditions. The main crystalline phases of the six samples are mullite (PDF# 15-0776) and quartz (PDF# 85-0930). The diffraction peaks located at 26.3°and 26.6°are attributed to the (210) facet of mullite phase and (011) facet of quartz phase, respectively. No diffraction peaks attributed to MnO_2_ were observed, probably due to the tiny amount of MnO_2_. Meanwhile, compared to the other five samples, the diffraction peak of M-0-1150 located at 26.6° slightly shifted to the direction of the low degree by 0.3°, indicating that the cell parameters had become larger. As no obvious difference in the diffraction peak positions between the undoped ceramic sample sintered at 1200 °C and the MnO_2_-doped counterparts sintered at 1150 °C was observed, we speculated that the added MnO_2_ effectively suppressed the abnormal growth of grains and the resulting formation of a large number of pores caused by a lower sintering temperature.

**Figure 2:**
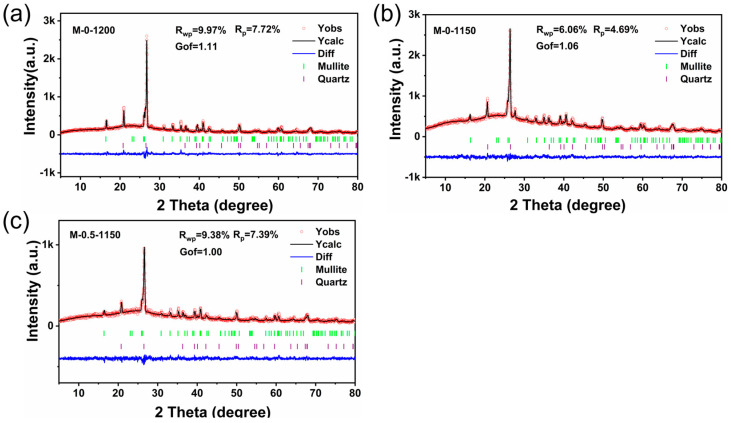
Rietveld refinement of the XRD patterns for (**a**) M-0-1200, (**b**) M-0-1150, and (**c**) M-0.5-1150.

Based on the obtained XRD patterns, the Rietveld method was employed to perform the crystal structure refinement. As displayed in Figure 2a–c, the calculated XRD patterns are in good agreement with the experimental patterns, indicated by the smooth difference curves (Diff). Meanwhile, the R_wp_ and R_p_ values less than 10% and the Gof values between 1 and 2 confirmed the reliability of the refinement results. Table 2 lists the refined cell parameters for the M-0-1200, M-0-1150, and M-0.5-1150 samples. The proportions of the mullite phase in the three ceramic samples are 43.39 wt.%, 41.45 wt.%, 44.40 wt.%, respectively, indicating that lower sintering temperatures adversely affected the formation of the mullite phase and this situation can be improved by the MnO_2_ additive. It’s well known that more Mullite phase in aluminosilicate ceramics benefits for the enhancement in mechanical strength. Moreover, a certain extent of the increase in the cell parameters of M-0-1200 was observed, relative to those of M-0-1150. In terms of the cell volume, lowering the sintering temperate by 50 °C rendered the cell volume to increase from 168.5400 Å^3^ to 169.7880 Å^3^. And it was lowered to 168.9500 Å^3^ after the addition of 0.5 wt.% of MnO_2_, getting close to that of the M-0-1200 sample. Theoretically, the smaller the cell volume, the smaller the porosity.

### 3.2. Thermogravimetric Analysis (TGA) and Differential Scanning Calorimetry (DSC)

**Figure 3:**
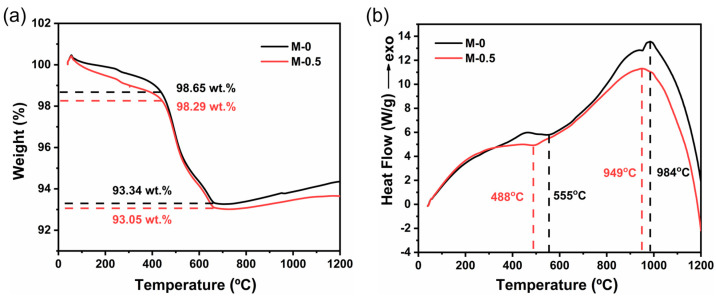
(**a**) TGA and (**b**) DSC curves of M-0 and M-0.5 samples.

The TGA and DSC analysis of the M-0 and M-0.5 samples were performed and the results are shown in Figure 3. In Figure 3a, weight loss occurred in the in the M-0.5 sample in the temperature range of 30–500 °C. The moisture residue in the ceramic body was converted to water vapor and removed rapidly, possibly accompanied by the elimination of some impurities, in a thermal environment. For the DSC curves in Figure 3b, an endothermic peak appeared at ~488 °C for the M-0.5 sample, while the endothermic peak for the M-0 sample appeared at ~550 °C. The emergence of the endothermic peak probably resulted from the removal of the crystal water in the ceramic body. The shift of the endothermic peak to the direction of a low temperature suggested that the MnO_2_ doping facilitated the removal of crystal water in the sintering of the aluminosilicate ceramic body. Moreover, owing to the crystallization process, an exothermic peak for the M-0.5 sample appeared at ~949 °C, lower by 35 °C than that of the M-0 sample, which suggested that the MnO_2_ in the ceramic body made the crystallization start to occur at a lower temperature. Based on these results, we deduced that enough MnO_2_ additive promoted the rapid elimination of moisture residue and crystal water in the ceramic body sintered at a lower temperature and thus reduced the total pore amount and volume. It suppressed the abnormal growth of grains during the sintering process and improved the densification of ceramics [32].

### 3.3. Composition Analysis

**Figure 4:**
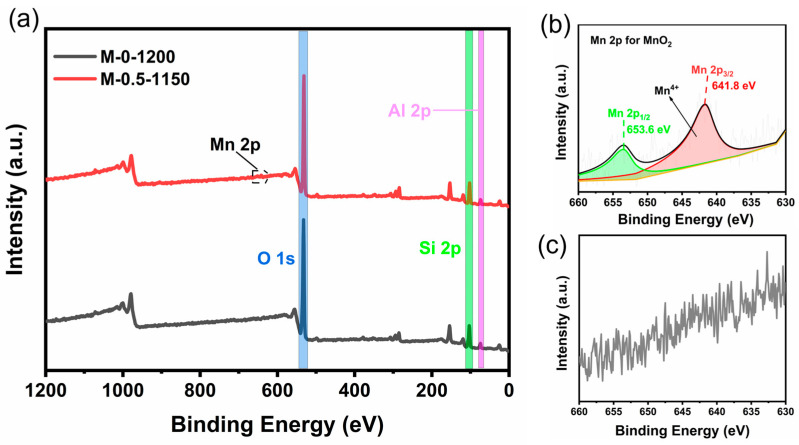
(**a**) Full XPS spectra of M-0.5-1150 and M-0-1200 samples. (**b**) High-resolution XPS spectrum of the Mn 2p core level for M-0.5-1150 sample. (**c**) High-resolution XPS spectrum of the Mn 2p core level for M-0-1200 sample.

Because of the detection limit of XRD and the low amount of addition (<0.5 wt.%), the MnO_2_ phase that possibly exists in the ceramic sample may not be detected. Thereby, X-ray photoelectron spectroscopy (XPS) was used to analyze the valence state of the Mn element in the prepared sample to determine the existing form of Mn; the results are shown in Figure 4. Figure 4a presents the Si2p, Al2p, and O1s signals in the M-0-1200 and M-0.5-1150 samples. No signal belonging to the Mn element was observed for the M-0-1200 sample (Figure 4c), while two peaks located at 653.6 eV and 641.8 eV, ascribed to Mn (IV) 2p1/2 and 2p3/2, were observed for the Mn-0.5-1150 sample (Figure 4b). This result indicates the existence of MnO_2_.

XRF was employed to quantify the MnO_2_ in the as-prepared ceramic samples. As listed in Table 3, the contents of the main components, Al_2_O_3_ and SiO_2_, are similar for all the samples. Approximately 0.08 wt.% of MnO_2_ exists in the undoped ceramic samples, likely coming from the aluminosilicate ceramic slurry itself provided by the company. For the doped ceramic samples, the measured content of MnO_2_ gradually increases from 0.18 to 0.89 wt.%, with the amount added increasing from 0.05 to 0.5 wt.%.

### 3.4. Effect of MnO_2_ on Ceramic Macroscopic Properties

**Figure 5:**
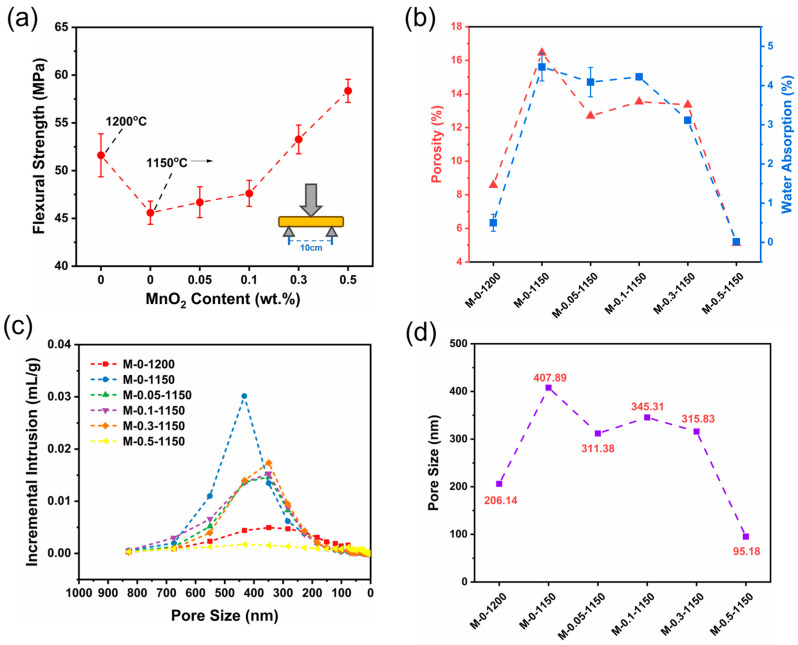
(**a**) Three-point flexural strength, (**b**) porosity factor and water absorption, (**c**) the relationship between pore size and incremental intrusion of Hg, and (**d**) pore size of as-prepared ceramic samples.

For the impact of the MnO_2_ addition, the flexural strength and water absorption of the ceramic samples were characterized by a three-point bending test and mercury intrusion porosimetry, respectively. Figure 5a shows that without MnO_2_ addition, the flexural strength of sample decreased by 11.65%, from 51.61 MPa to 45.59 MPa, upon the reduction in the sintering temperature from 1200 °C to 1150 °C. After MnO_2_ doping and sintering at 1150 °C, the flexural strength of the sample was enhanced gradually with the increase in the MnO_2_ doping amount. The 0.3 wt.% of MnO_2_ addition enabled the flexural strength of M-0.3-1150 to reach 53.27 MPa, exceeding that of M-0-1200. Further increasing the MnO_2_ content to 0.5 wt.% endowed the M-0.5-1150 sample with a flexural strength of 58.36 MPa, higher than that of M-0-1200 by 13.08%. The experimental data show that the flexural strength of MnO_2_ sintered at 1150 °C with an addition of 0.5 wt.% exceeds that of the undoped sample sintered at 1200 °C. Based on these results, we speculated that the MnO_2_ doping made the ceramic sample become more densified and possess more grain boundaries, and the latter largely avoided the excessive growth of cracks, absorbed more energy, and acted as a buffer against cracks. MnO_2_ is beneficial to stop the abnormal growth of grains caused by lower sintering temperatures [33].

Figure 5b displays the porosity and water absorption of ceramic samples as a function of the MnO_2_ doping amount. In the 0–0.3 wt.% of MnO_2_ doping range, both the porosity and water absorption of the samples sintered at 1150 °C were remarkably higher than those of the M-0-1200 sample. It is not until the doping amount of MnO_2_ reaches 0.5 wt.% that the porosity (5.12%) and water adsorption (0.05%) of the samples sintering at 1150 °C become lower than those of the M-0-1200 sample. Because of the increase in the addition of MnO_2_, more adsorbed water and crystallized water are excluded, reducing the generation of pores and thus increasing the densification. Figure 5c displays the relationship between the pore size and incremental intrusion of Hg. The change trend in the intrusion of Hg with pore size was basically in line with that of porosity. In the whole range of pore size (0~800 nm), the integrated area of M-0-1150 is remarkably larger than those of the other samples, indicating that the M-0-1150 sample has the largest pore volume (Table 4). For the samples sintered at 1150 °C, the intrusion of Hg decreased upon the addition of MnO_2_. The content of MnO_2_ added within 0–0.1 wt.% slightly decreased the intrusion of Hg, but no obvious intrusion of Hg was observed for the M-0.5-1150 sample with 0.5 wt.% of MnO_2_ added content. This indicated that the M-0.5-1150 sample possesses the lowest pore volume among all the prepared ceramic samples. This is likely because MnO_2_, as the sintering aid, was transformed into the liquid phase in the sintering process; the liquid-phase MnO_2_ flowed into pores, expelling the gas in the pores and filling the pores. The insufficient addition of MnO_2_ rendered this phenomenon weakened. As listed in Table 4, the decreased trends of apparent density and pore volume occurred to the ceramic samples with MnO_2_ doping. And the M-0.5-1150 sample has the lowest apparent density, indicating that a number of closed pores formed inside it [34]. This is probably because the liquid-phase MnO_2_ forming in the sintering process filled the gaps between grains, generating more closed pores. Also, this is the reason why M-0.5-1150 has a remarkably low water absorption.

Pore size is one of the most important factors affecting the performance of ceramics, and the pore sizes of the six prepared samples are shown in Figure 5d. The average pore sizes of M-0-1200 and M-0-1150 were 206.14 nm and 407.89 nm, respectively. The decline of 50 °C in sintering temperature caused a 2-fold increase in pore size. An additional 0.5 wt.% amount of MnO_2_ decreased the pore size of the M-0.5-1150 sample by 1/2 and 3/4, compared to those of the M-0-1200 and M-0-1150 samples, respectively, validating the effectiveness of MnO_2_ doping in the pore size reduction in ceramic samples. This is also the reason why MnO_2_ doping could enhance the density and flexural strength of ceramics.

### 3.5. Surface Chemical Species and Morphology Analysis

**Figure 6:**
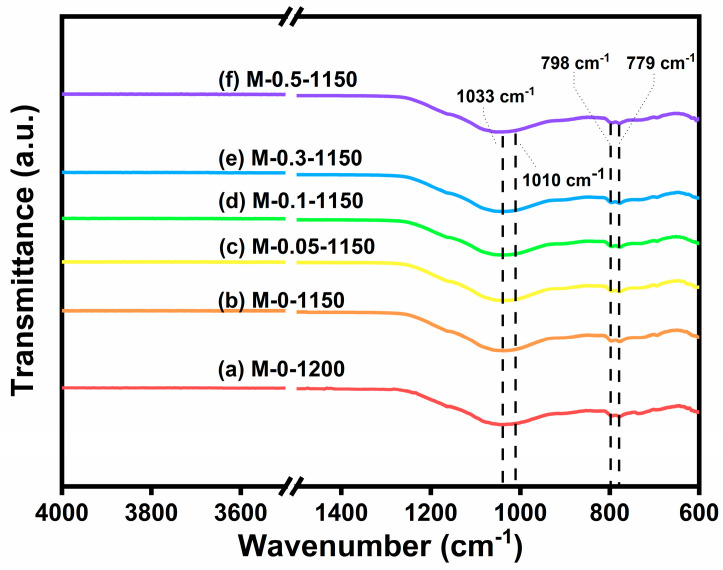
The FTIR spectra of (a) M-0 sintering at 1200 °C and (b–f) M-0, M-0.05, M-0.1, M-0.3, M-0.5 sintering at 1150 °C.

As a conventional structural characterization method, FTIR spectroscopy can identify the functional groups and vibration modes in materials. In silicate crystals, the composition contains a variety of other cations in addition to silicon and oxygen, which may result in the occurrence of an isomorphous replacement. Thus, FTIR characterization was conducted to figure out whether manganese ions replaced aluminum ions in the aluminosilicate crystals after the addition of MnO_2_ [35]. Figure 6 shows the FTIR spectra of the six sintered ceramic samples. The peaks appearing at 779 and 798 cm^−1^ are from the quartz phase [36]. And the absorption band at 1033 and 1010 cm^−1^ arise from the vibrations of Si-O-Si and Si-O-Al, respectively [14]. No noticeable peak that arose from the MnO_2_ doping appeared, indicating that 0–0.5 wt.% MnO_2_ doping did not lead to manganese ion replacement during crystallization.

**Figure 7:**
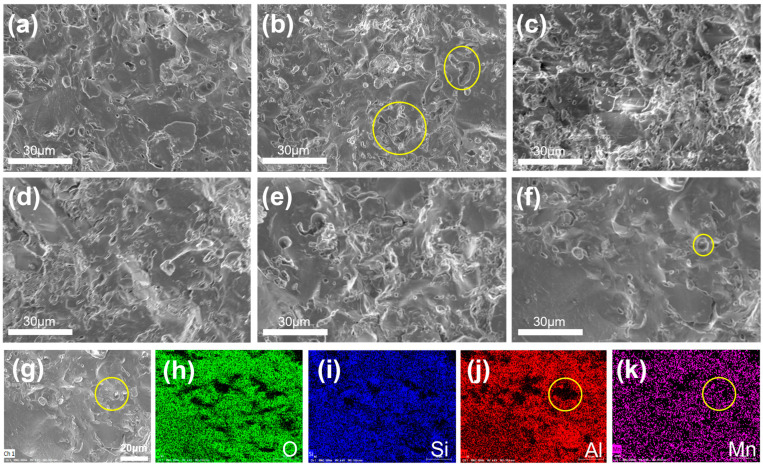
(**a**–**f**) SEM images of M-0-1200,M-0-1150, M-0.05-1150, M-0.1-1150, M-0.3-1150 and M-0.5-1150. (**g**) Field emission SEM images of M-0.5-1150 and element mapping results of (**h**) O, (**i**) Si, (**j**) Al, (**k**) Mn signals. The pores are marked by yellow circle.

SEM and EDS characterizations were performed to observe the pores in the microstructure and densification degree of the samples (Figure 7). Figure 7a–f shows the SEM images of the aluminosilicate ceramics doped with different contents of MnO_2_. Of them, M-0-1150 has the most pores (Figure 7b), M-0.5-1150 has the least pores (Figure 7f) and the M-0.5-1150 sample possesses the smallest pore size, compared to other five samples; this is probably ascribed to the suppressing effect on the pore formation in the sintering process by the sufficient MnO_2_ additive. This again proves that the addition of MnO_2_ to aluminosilicate ceramics can promote their densification. As displayed in Figure 7j-k, the EDS mapping results showed that the region around the pores was rich in the Mn element, instead of the Al element. It implied that MnO_2_ may transform into liquid and flow into the pores in the sintering process, which promoted the gas removal and reduction in pore size. Additionally, the uniform distribution of the Mn element in the samples validated that the preparation of such aluminosilicate ceramics can be achieved through simple mechanical stirring and the common sintering process.

## 4. Conclusions

In this study, we prepared MnO_2_-doped aluminosilicate ceramics and disclosed that a 0.3 wt.% addition of MnO_2_ can sufficiently make the aluminosilicate ceramics sintered at 1150 °C possess identical flexural strength as the undoped counterpart sintered at 1200 °C (M-0-1200 sample). The lowest water absorption reaches 0.05%, merely one-tenth of the water absorption for the M-0-1200 ceramic sample. The added MnO_2_ can decrease both the pore size and total pore volume, and enhance the sintering densification; this is probably because the MnO_2_ transforms into a liquid phase in the sintering process and flows into the gap between grains which removes the gas inside the pores and fills the pores, suppressing the generation of pores and the abnormal growth of grains. Also, this study demonstrated a facile method, doping with MnO_2_ using mechanical stirring at room temperature, to lower the water absorption, enhance the flexural strength, and reduce the sintering temperature concurrently, which has a promising value for practical ceramic production.

## Figures and Tables

**Table 1:** Chemical composition of raw materials.

Oxides	SiO_2_	Al_2_O_3_	MnO_2_	K_2_O	Fe_2_O_3_	CaO	Other
Content (wt.%)	60~65	20~25	0~0.1	2~4	1~2	0.5~1	2.9~16.5

**Table 2:** The cell parameters of the ceramic samples.

	Samples	M-0-1200	M-0-1150	M-0.5-1150
Phase 1	Quartz (wt.%)	56.61	58.55	55.60
	Space Group	P3121	P3121	P3121
Cell Parameters	a (Å)	4.9318 (61)	4.9417 (67)	4.9421(13)
	b (Å)	4.9318 (61)	4.9417 (67)	4.9421(13)
	c (Å)	5.4216 (84)	5.4368 (10)	5.4239 (19)
	V (Å^3^)	114.1990 (33)	114.9780 (38)	114.7280 (73)
Phase 2	Mullite (wt.%)	43.39	41.45	44.40
	Space Group	Pbam	Pbam	Pbam
Cell Parameters	a (Å)	7.5652 (12)	7.5840 (15)	7.5674 (23)
	b (Å)	7.7073 (12)	7.7279 (16)	7.7158 (25)
	c (Å)	2.8906 (43)	2.8970 (50)	2.8935 (10)
	V (Å^3^)	168.5400 (45)	169.7880 (57)	168.9500 (95)

**Table 3:** Chemical compositions of as-prepared ceramic samples ^a^.

Sample	Al_2_O_3_ (wt.%)	SiO_2_ (wt.%)	MnO_2_ (wt.%)	Others (wt.%)
M-0-1200	25.41	66.00	0.07	8.53
M-0-1150	25.38	63.39	0.09	11.14
M-0.05-1150	26.43	64.44	0.18	8.96
M-0.1-1150	23.31	63.39	0.54	12.77
M-0.3-1150	24.32	64.15	0.84	10.69
M-0.5-1150	26.33	64.14	0.89	8.64

^a^ Measured using XRF.

**Table 4:** Physical properties of the as-prepared ceramic samples ^a^.

Sample	Apparent Density (g/mL)	Pore Volume (mL/g)
M-0-1200	2.4799	0.0378
M-0-1150	2.5442	0.0774
M-0.05-1150	2.4931	0.0583
M-0.1-1150	2.5054	0.0625
M-0.3-1150	2.4978	0.0617
M-0.5-1150	2.4205	0.0223

^a^ Data from MIP tests.

## Data Availability

Data are contained within the article.
